# Associations of Uric Acid With Liver Steatosis and Fibrosis Applying Vibration Controlled Transient Elastography in the United States: A Nationwide Cross-Section Study

**DOI:** 10.3389/fendo.2022.930224

**Published:** 2022-06-23

**Authors:** Hualin Duan, Rong Zhang, Xingying Chen, Genfeng Yu, Cheng Song, Yuqi Jiang, Yajun He, Heng Wan, Jie Shen

**Affiliations:** ^1^ Department of Endocrinology and Metabolism, Shunde Hospital, Southern Medical University (The First People’s Hospital of Shunde Foshan), Foshan, China; ^2^ Department of Endocinology & Metabolic Diseases, Affiliated Hospital of Xiangnan University, Chenzhou, China; ^3^ Department of Endocrinology, The Third Affiliated Hospital of Southern Medical University, Guangzhou, China

**Keywords:** liver steatosis, liver fibrosis, controlled attenuation parameter, liver stiffness measurement, uric acid, NHANES

## Abstract

**Background and objective:**

Serum uric acid (UA) is related to many metabolic diseases. However, the association of UA with liver diseases was not very clear. The objective of this study is to clarify the relationship of UA with liver steatosis and fibrosis.

**Methods:**

This is a cross-sectional study of 4364 people of National Health and Nutrition Examination Survey (NHANES) 2017-2018. Liver steatosis and fibrosis were assessed by controlled attenuation parameter (CAP) and liver stiffness measurements (LSM) using Vibration-controlled transient elastography (VCTE). Linear and logistic regressions were performed.

**Results:**

After adjusting for potential confounders, UA levels were associated with the prevalence of liver steatosis [OR=2.097 (95%CI: 1.245, 3.534)] and liver fibrosis [OR=2.983 (95%CI: 1.797, 4.952)]. Furthermore, the results were consistent in the subgroup analyses of males and females.

**Conclusions:**

UA levels were positively associated with the prevalence of liver steatosis and fibrosis.

## 1 Introduction

The global burden of chronic liver disease (CLD) and its complications is substantial. More than 44,000 deaths in the United States and 2 million deaths worldwide each year are attributable to CLD and cirrhosis (1, 2). Liver steatosis and liver fibrosis are the common pathophysiological processes of many liver diseases. Thus, they are often used as indicators to evaluate the development and prognosis of CLD and cirrhosis. For example, liver steatosis is the primary diagnostic criterion of Nonalcoholic fatty liver disease (NAFLD) (3). Liver fibrosis as a precursor of cirrhosis is a pivotal pathological process in the evolution of all chronic liver diseases to cirrhosis (4). Therefore, it is of great significance to find possible risk factors related to hepatic steatosis and fibrosis, which may allow us to detect liver abnormalities earlier and provide ideas for the prevention and treatment of hepatic steatosis and fibrosis.

Serum uric acid (UA) is a metabolite of purine and is a recognized important cause of hyperuricemia and kidney stones (5). Although previous studies have investigated the associations between UA levels and the prevalence of CLD (6), the relationship between UA and liver steatosis and fibrosis has been controversial. One early study of Koreans found higher UA levels were independently associated with hepatic steatosis assessed by ultrasonography (7). However, one recent study of 541 participants showed UA levels in women were not associated with the risk of developing hepatic steatosis (8). Meanwhile, there were few large sample size studies about the relationship of UA and liver fibrosis.

Moreover, many previous large-scale epidemiological studies on the relationship between UA and hepatic steatosis were diagnosed by ultrasonography. However, vibration controlled transient elastography (VCTE) was used in this study. VCTE has become a more common tool to aid in the management of patients with chronic liver disease (9). The controlled attenuation parameter (CAP) and liver stiffness measurements (LSM) assessed by VCTE, reflected the liver steatosis and fibrosis respectively (10). Compared with ordinary B-ultrasound, it can quantitatively evaluate the steatosis and fibrosis conditions of the liver. It also has higher accuracy, when compared with nonproprietary serum-based fibrosis markers (11).

Thus, the data of National Health and Nutrition Examination Survey (NHANES) was analyzed to clarify the relationship of serum uric acid with the prevalence of liver steatosis and fibrosis *via* a non-invasive, quantitative and high-accuracy manner.

## 2 Material and Methods

### 2.1 Study Participants

**Figure 1:**
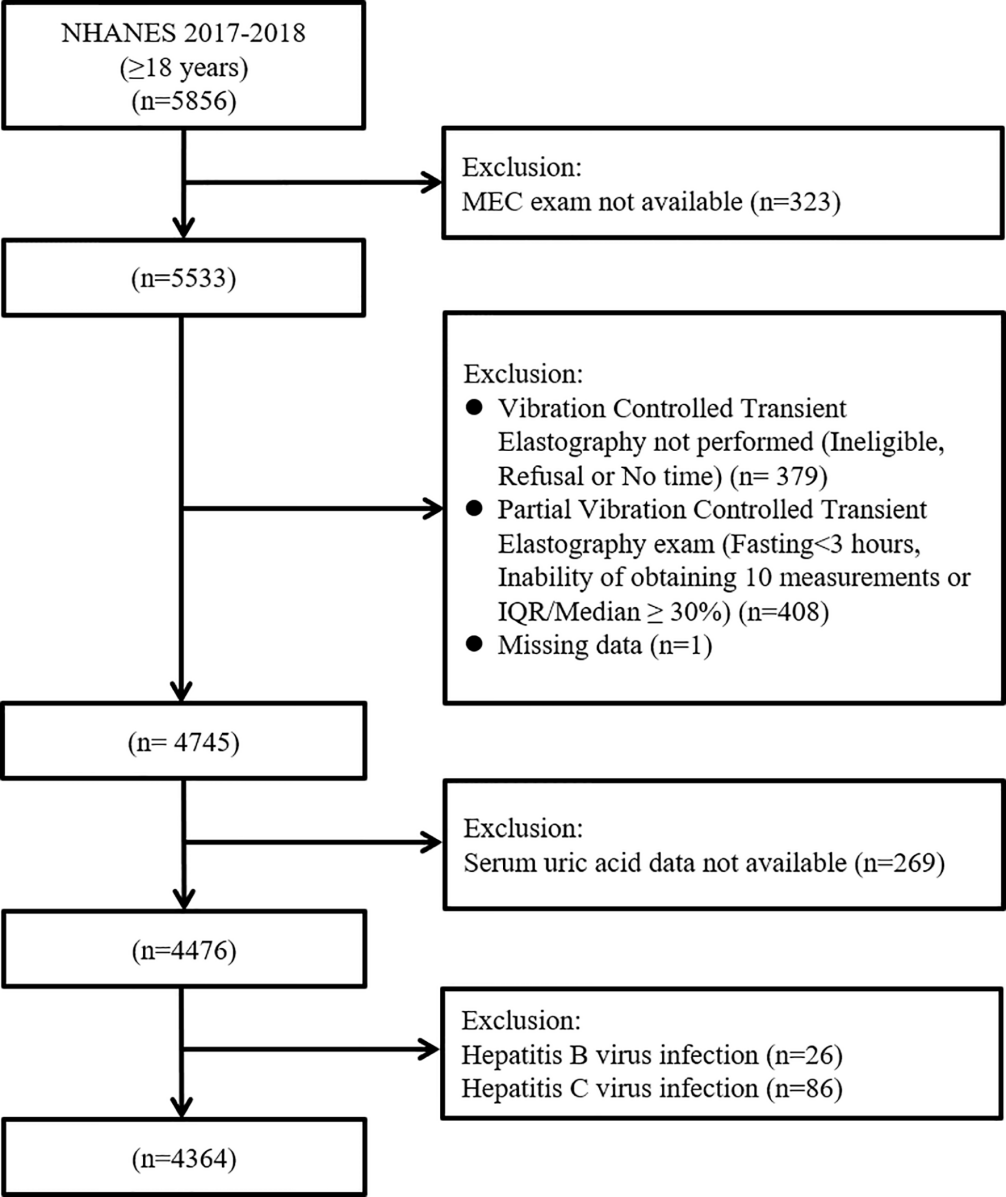
Flow-chart of the study participants selected from National Health and Nutrition Examination Survey (NHANES) 2017–2018.

This is an analysis of data from the participants of the 2017–2018 cycle of the National Health and Nutrition Examination Survey (NHANES). The NHANES is a major program of the National Center for Health Statistics, which is part of the Centers for Disease Control and Prevention. It is a nationally representative cross-sectional study which consists of interview, examination, and laboratory data collected from complex multistage, stratified, clustered probability samples with oversampling of civilian noninstitutionalized US population (12). There were 5856 participants aged ≥18 years in the 2017-2018 cycle of the NHANES. Initially, 323 individuals, who have not attended a mobile examination center visit, were excluded. Then, we excluded 379 patients without vibration controlled transient elastography (VCTE) exam, 408 patients with the VCTE exam as partial and 1 patient with missing CAP data. We additionally excluded 269 participants whose serum uric acid data were missing. Finally, we excluded 26 participants with HBV and 86 participants with HCV, leading to a final sample pool of 4364 participants with complete data. (**Figure 1**).

### 2.2 Measurements and Definition of Variables

#### 2.2.1 Serum Uric Acid

Serum specimens are collected, processed and stored in the mobile examination center (MEC), then shipped to the University of Minnesota – Advanced Research Diagnostics Laboratory, Minneapolis, MN for analysis (13). In the test, uric acid is oxidized by uricase. Then the peroxide produced from this reaction is acted upon by peroxidase with the presence of aminophenazone to produce a measurable colored product. It is a two-point, endpoint reaction, with the measurement of light absorbance value at 546 nm (secondary wavelength 700 nm) (13).

#### 2.2.2 Vibration Controlled Transient Elastography

VCTE measures the speed of mechanically generated shear waves across the liver to obtain a liver stiffness measurement (LSM), a marker of hepatic fibrosis. VCTE can also simultaneously measure the attenuation of the ultrasound signal through the liver to obtain the controlled attenuation parameter (CAP), which is a marker of hepatic steatosis (10). VCTE using FibroScan 502 V2 Touch (Echosens) was performed on eligible participants. Each participant was measured 30 times using either a medium (M) or large (XL) probe. The medium probe was applied firstly, unless the manufacturer’s instructions recommend that the XL probe should be used. Liver status imaging with the subject lying in supine position were obtained. Examinations were considered reliable if (i) participants fasted at least 3 hours before the exam, (ii) 10 or more complete LSM were performed, (iii) the interquartile range/median of LSM < 30% (14). CAP≥274 dB/m was considered as a indicator of liver steatosis, LSM≥8kPa indicated the presence of liver fibrosis according to the literature (15, 16). For sensitivity analysis, 248 dB/m and 7 kPa as thresholds for liver steatosis and liver fibrosis respectively, suggested by other literature, were used (17, 18).

#### 2.2.3 Covariates

Demographic information such as sex, age, race and questionnaire data like alcohol consumptions, smoking status and menopausal status were obtained in household interviews. Physical examinations (includes blood pressure, body measures and vibration controlled transient elastography) and glycohemoglobin (HbA1c) data were obtained in the mobile examination center (MEC) and cooperative laboratory. Methods for measuring urinary albumin to creatinine ratio (UACR) and serum creatinine (SCr) have been reported in detail elsewhere (19–21). Race was self-reported as Mexican American, other Hispanic, non-Hispanic White, non-Hispanic Black, or other races. Estimates of alcohol consumption were based on information provided by the respondent about the amount and frequency of alcohol consumption during the previous year. The amount of consumed alcohol was reported in standard drinks and converted to grams using a multiplication factor of 14. It was considered as alcohol abuse, if the consumed amount of alcohol ≥30g/day for male and ≥20g/day for female (22, 23). Smoking status were divided into never, ever and current smoking groups according to self-report. The usage of diuretics and pioglitazone was obtained through a questionnaire (24). Body measures including height (m), weight (kg), and body mass index (BMI), calculated as weight in kilograms divided by height in meters squared, were categorized into underweight (BMI < 18.5 kg/m2), normal (BMI 18.5-24.9kg/m2), overweight (BMI 25-29.9 kg/m2) and obese (BMI ≥ 30 kg/m2) groups. Blood pressure was measured 3 times by certified physicians and the average of these measurements was chosen as the representative value. The percentage of HbA1c was determined by high pressure liquid chromatography (25). Hepatitis C virus infection was indicated by presence of viral RNA and/or a confirmed antibody test and hepatitis B virus infection as a positive surface antigen test (15, 26).

#### 2.2.4 Statistical Analysis

All analyses were conducted using IBM SPSS Statistics 26 (SPSS Inc., Armonk, New York, USA) and the Complex Samples module. A two-tailed P value < 0.05 suggests statistically significance. Because of the complex design of NHANES, proper weights should be used in making estimates that are representative of the U.S. civilian non-institutionalized population. In this study, Full sample 2-year MEC exam weights were used.

First of all, baseline characteristics of participants are presented as the means (SE) (continuous variable) or percentages (categorical variable). The UA levels were divided into four quartiles in total participants, males and females, respectively. CAP and LSM were transformed to achieve a normal logarithmic distribution. linear regression analysis was performed to analyze the association of serum uric acid with Ln CAP and Ln LSM, and B regression coefficients were obtained (95% confidence intervals [CI]). Then logistic regression analysis was applied to analyze the association of UA with the prevalence of liver steatosis and liver fibrosis, and odds ratios (ORs) were obtained (95% CI). Furthermore, to investigate whether there is sex specificity, a sex-stratified analysis was performed. For the total participants, the adjusted model including sex, age, race, smoking status, BMI (underweight, normal weight, overweight, obese), alcohol abuse, HbA1c and systolic pressure, UACR, SCr, usage of diuretics and usage of pioglitazone. In the subgroup analyses of males and females, we adjusted all confounders in the model except for sex. To determine whether menopausal status affected the association of UA with CAP and LSM, female samples were divided into premenopausal and postmenopausal groups, followed by linear and logistic regression analysis, in the supplementary materials. For menopausal status, women were categorized as postmenopausal if they had both ovaries removed or if periods had stopped because of menopause or if menopausal status unknown but ≥ 50 years. Otherwise, they were categorized as premenopausal (27). Finally, Sensitivity analysis was performed in the supplementary materials with different thresholds for liver steatosis and fibrosis.

### 2.3 Ethics Considerations

The original survey was approved by the National Center for Health Statistics (NCHS) Research Ethics Review Board, informed consent was obtained from each patient included in the study; the study protocol conforms to the ethical guidelines of the Declaration of Helsinki.

## 3 Results

### 3.1 General Description

**Table 1** describes the basic characteristics of US adult, including sex, age, race, smoking status, BMI, alcohol abuse, HbA1c, systolic pressure, serum uric acid, UACR, SCr, usage of diuretics and usage of pioglitazone according to CAP and LSM respectively. Among the 4364 participants, there were 1918 with liver steatosis and 419 with liver fibrosis. Compared to people without liver steatosis, people with liver steatosis are more likely to be elders and males, with higher HbA1c, systolic pressure, serum uric acid, UACR and SCr levels and higher prevalence of smoking, obesity, alcohol abuse and usage of diuretics. Compared to people without liver fibrosis, people with liver fibrosis are more likely to be elders and males, with higher HbA1c, systolic pressure, serum uric acid, UACR and SCr levels and higher prevalence of smoking, obesity and usage of diuretics. **Table 2** describes the basic characteristics of US adults according to the quartiles of serum UA. *P* for trend was presented. There were also significant trends of sex, age, smoking status, BMI, systolic pressure, CAP, LSM, SCr and usage of diuretics among the populations with different quartiles of serum UA levels.

**Table 1:** Basic characteristics of US adults according to CAP and LSM from NHANES 2017–2018.

	CAP, dB/m		LSM, kPa	
	<274	≥274	<8	≥8
**N**	2446	1918	3945	419
**Sex, %**				
Male	44.0 ± 1.3	55.5 ± 1.4	48.1 ± 1.0	57.7 ± 3.1
Female	56.0 ± 1.3	44.5 ± 1.4	51.9 ± 1.0	42.3 ± 3.1
**Age, years**	44.2 ± 0.7	50.7 ± 0.7	46.5 ± 0.7	51.3 ± 1.3
**Race, %**				
Mexican American	7.0 ± 1.3	12.8 ± 2.4	9.4 ± 1.8	11.2 ± 2.1
Other Hispanic	7.6 ± 1.0	6.3 ± 0.8	7.0 ± 0.9	7.9 ± 1.6
Non-Hispanic white	62.7 ± 2.7	62.2 ± 2.7	62.6 ± 2.5	61.1 ± 4.1
Non-Hispanic black	12.2 ± 1.7	8.1 ± 1.4	10.5 ± 1.5	9.9 ± 2.5
Other	10.6 ± 1.4	10.4 ± 1.3	10.6 ± 1.2	9.9 ± 2.1
**Smoking status, %**				
Never	61.9 ± 1.9	57.3 ± 1.8	60.5 ± 1.5	53.9 ± 3.0
Ever	21.5 ± 1.3	28.0 ± 1.8	23.6 ± 1.0	31.9 ± 3.1
Current	16.6 ± 1.4	14.7 ± 1.3	15.9 ± 1.2	14.2 ± 2.0
**BMI, %**				
Underweight	2.4 ± 0.3	0.2 ± 0.1	1.5 ± 0.2	0.1 ± 0.1
Normal	40.6 ± 2.1	6.6 ± 1.0	27.6 ± 1.6	10.2 ± 2.9
Overweight	34.3 ± 1.3	27.3 ± 2.1	32.8 ± 1.4	13.2 ± 2.1
Obese	22.9 ± 1.8	65.9 ± 2.8	38.0 ± 2.2	76.4 ± 3.7
**Alcohol abuse, %**	6.1 ± 0.7	8.0 ± 0.9	7.0 ± 0.7	5.1 ± 1.9
**HbA1c, %**	5.4 ± 0.0	5.9 ± 0.0	5.6 ± 0.0	6.3 ± 0.1
**Systolic, mmHg**	119.2 ± 0.5	124.2 ± 0.6	121.0 ± 0.5	125.8 ± 1.1
**UA, mg/dL**	5.4 ± 0.0	5.9 ± 0.0	5.3 ± 0.0	6.0 ± 0.1
**UACR, mg/g**	28.8 ± 5.2	36.2 ± 4.5	28.0 ± 3.2	75.4 ± 19.5
**SCr, mg/dL**	0.87 ± 0.0	0.89 ± 0.0	0.87 ± 0.0	0.92 ± 0.0
**Usage of diuretics, %**	3.5 ± 0.5	10.1 ± 1.1	5.7 ± 0.5	12.3 ± 2.0
**Usage of Pioglitazone, %**	0.5 ± 0.2	0.4 ± 0.1	0.5 ± 0.1	0.0 ± 0.0

Data are expressed as weighted proportions ( ± standard error [SE]) for categorical variables and as weighted means ± SE for continuous variables.

BMI, body mass index; CAP, controlled attenuation parameter; HbA1c, Glycohemoglobin; LSM, liver stiffness measurement; SCr, serum creatinine; UA, uric acid; UACR, albumin to creatinine ratio.

**Table 2:** Basic characteristics of US adults according to serum uric acid from NHANES 2017–2018.

	UA, mg/dL				*P for trend*
	Q1(≤4.4)	Q2(4.4~5.3)	Q3(5.3~6.4)	Q4(>6.4)	
N	1164	1058	1126	1016	
**Sex, %**					**<0.001**
Male	17.1 ± 1.6	40.4 ± 2.7	66.2 ± 1.5	78.6 ± 2.2	
Female	82.9 ± 1.6	59.6 ± 2.7	33.8 ± 1.5	21.4 ± 2.2	
**Age, years**	45.7 ± 0.8	45.2 ± 0.9	48.5 ± 0.7	48.4 ± 1.1	**0.003**
**Race, %**					0.124
Mexican American	10.6 ± 2.0	9.3 ± 1.9	8.9 ± 1.8	9.2 ± 1.9	
Other Hispanic	8.0 ± 1.4	7.2 ± 1.1	6.8 ± 1.0	6.0 ± 0.9	
Non-Hispanic white	62.1 ± 3.5	62.5 ± 3.4	65.5 ± 2.2	59.2 ± 2.8	
Non-Hispanic black	11.1 ± 1.6	9.9 ± 1.6	8.9 ± 1.8	12.4 ± 2.2	
Other	8.4 ± 1.2	11.1 ± 1.8	10.0 ± 1.4	13.2 ± 1.5	
**Smoking status, %**					**0.005**
Never	65.2 ± 2.0	60.1 ± 2.4	56.0 ± 2.4	57.9 ± 2.6	
Ever	17.7 ± 1.5	22.4 ± 1.6	29.0 ± 1.8	29.0 ± 2.3	
Current	17.1 ± 1.4	17.4 ± 2.1	15.0 ± 2.2	13.1 ± 1.4	
**BMI, %**					**<0.001**
Underweight	2.9 ± 0.6	1.4 ± 0.4	0.8 ± 0.4	0.3 ± 0.1	
Normal	40.4 ± 1.4	30.0 ± 3.3	19.6 ± 1.7	11.4 ± 1.4	
Overweight	28.7 ± 2.1	28.7 ± 2.8	34.7 ± 2.1	33.1 ± 2.7	
Obese	28.1 ± 2.1	39.8 ± 2.7	44.9 ± 3.0	55.1 ± 3.4	
**Alcohol abuse, %**	6.0 ± 1.4	5.5 ± 1.3	7.9 ± 1.4	8.1 ± 1.0	0.406
**Glycohemoglobin, %**	5.6 ± 0.0	5.6 ± 0.04	5.6 ± 0.0	5.7 ± 0.0	0.178
**Systolic, mmHg**	118.1 ± 1.1	121.1 ± 0.6	122.8 ± 0.7	124.1 ± 0.6	**0.001**
**CAP, dB/m**	239.9 ± 2.6	256.0 ± 3.1	271.7 ± 2.2	289.9 ± 2.9	**<0.001**
**LSM, kPa**	4.8 ± 0.1	5.6 ± 0.2	5.8 ± 0.3	6.4 ± 0.2	**<0.001**
**UACR, mg/g**	27.6 ± 6.0	22.3 ± 3.2	35.5 ± 10.1	44.3 ± 7.4	0.174
**SCr, mg/dL**	0.76 ± 0.0	0.83 ± 0.0	0.93 ± 0.0	1.02 ± 0.0	**<0.001**
**Usage of diuretics, %**	2.4 ± 0.5	3.9 ± 0.5	7.6 ± 1.1	12.4 ± 1.3	**<0.001**
**Usage of Pioglitazone, %**	0.1 ± 0.1	0.3 ± 0.1	0.7 ± 0.2	0.6 ± 0.5	0.130

Data are expressed as weighted proportions ( ± standard error [SE]) for categorical variables and as weighted means ± SE for continuous variables.

BMI, body mass index; CAP, controlled attenuation parameter; HbA1c, Glycohemoglobin; LSM, liver stiffness measurement; SCr, serum creatinine; UA, uric acid; UACR, albumin to creatinine ratio.

The bold indicates significance (P < 0.05).

### 3.2 The Associations Between UA With CAP

**Table 3** shows the associations between UA with CAP. Linear regression analyses showed that compared with the first quartile of UA, individuals in the fourth quartile had the highest B for Ln CAP in total participants [B=0.082 (95%CI: 0.046, 0.118)], males [B=0.097 (95%CI: 0.056, 0.138)] and females [B=0.099 (95%CI: 0.049, 0.149)]. Logistic regression analyses showed that serum UA levels were associated with the prevalence of liver steatosis in total participants [OR=2.097 (95%CI: 1.245, 3.534)], males [OR=2.231 (95%CI: 1.158, 4.299)] and females [OR=2.803 (95%CI: 1.391, 5.646)]. Adjusted model included sex, age, race, smoking status, BMI, alcohol abuse, HbA1c, systolic pressure, UACR, SCr, usage of diuretics and usage of pioglitazone. Moreover, sensitivity analyses were conducted using another definition of steatosis as ≥ 248 dB/m. The results remained similar (**Supplementary Table 1**). In addition, we further analyzed the associations in females stratified by menopausal status. **Supplementary Table 2** shows the associations between UA with CAP of premenopausal and postmenopausal females. Linear regression analyses showed that compared with the first quartile of UA, individuals in the fourth quartile had the highest B for Ln CAP in premenopausal females [B=0.128 (95%CI: 0.067, 0.188)] and postmenopausal females [B=0.091 (95%CI: 0.036, 0.146)].

**Table 3:** The associations between UA with CAP.

	Ln CAP	CAP≥274dB/m
SUA, mg/dL	B (95%CI)	OR (95%CI)
Total		
Q1(≤4.4)	Reference	Reference
Q2(4.4~5.3)	0.012 (-0.022, 0.046)	1.256 (0.860, 1.834)
Q3(5.3~6.4)	**0.046 (0.005, 0.087)**	**1.677 (0.957, 2.937)**
Q4(>6.4)	**0.082 (0.046, 0.118)**	**2.097 (1.245, 3.534)**
Males		
Q1(≤5.1)	Reference	Reference
Q1(5.1~6.0)	**0.033 (0.001, 0.064)**	1.186 (0.801, 1.756)
Q3(6.0~6.9)	0.013 (-0.033, 0.058)	0.986 (0.620,1.568)
Q4(>6.9)	**0.097 (0.056, 0.138)**	**2.231 (1.158, 4.299)**
Females		
Q1(≤3.9)	Reference	Reference
Q2(3.9~4.7)	**0.039 (0.006, 0.071)**	1.483 (0.898, 2.448)
Q3(4.7~5.6)	0.047 (-0.004, 0.098)	1.532 (0.922, 2.546)
Q4(>5.6)	**0.099 (0.049, 0.149)**	**2.803 (1.391, 5.646)**

Data are expressed as odds ratio (95% Confidence interval [CI]).

Adjusted model included sex, age, race, smoking status, BMI, alcohol abuse, glycohemoglobin, systolic pressure, UACR, SCr, usage of diuretics and usage of pioglitazone. In the subgroup analyses of males and females, adjusted all confounders in the model except sex.

CAP, controlled attenuation parameter; SCr, serum creatinine; UA, uric acid; UACR, albumin to creatinine ratio.

The bold indicates significance (P < 0.05).

### 3.3 The Associations Between UA With LSM

**Table 4** shows the associations between UA with LSM. Linear regression analyses showed that compared with the first quartile of UA, individuals in the fourth quartile had the highest B for Ln LSM in total participants [B=0.097 (95%CI: 0.037, 0.157)], males [B=0.070 (95%CI: -0.002, 0.142)] and females [B=0.124 (95%CI: 0.047, 0.201)]. Logistic regression analyses showed that serum UA levels were associated with the prevalence of liver fibrosis in total participants [(OR=2.983 (95%CI: 1.797, 4.952)], males [OR=1.777 (95%CI: 0.967, 3.267)] and females [OR=5.204 (95%CI: 2.148, 12.605)]. Adjusted model included sex, age, race, smoking status, BMI, alcohol abuse, HbA1c, systolic pressure, UACR, SCr, usage of diuretics and usage of pioglitazone. Furthermore, sensitivity analyses were conducted using another definition of fibrosis as ≥ 7 kPa. The results remained similar (**Supplementary Table 1**). In addition, the associations were also analyzed for females stratified by menopausal status. **Supplementary Table 3** shows the associations between UA with LSM in premenopausal and postmenopausal females. Logistic regression analyses showed that serum UA levels were associated with the prevalence of liver fibrosis in premenopausal females [OR=6.670 (95%CI: 1.355, 32.836)] and postmenopausal females [OR=4.613 (95%CI: 1.521, 13.986)].

**Table 4:** The associations between UA with LSM.

	Ln LSM	LSM≥8kPa
SUA, mg/dL	B (95%CI)	OR (95%CI)
Total		
Q1(≤4.4)	Reference	Reference
Q2(4.4~5.3)	0.034 (-0.006, 0.075)	**2.055 (1.027, 4.111)**
Q3(5.3~6.4)	0.059 (-0.014, 0.133)	1.725 (0.808, 3.686)
Q4(>6.4)	**0.097 (0.037, 0.157)**	**2.983 (1.797, 4.952)**
Males		
Q1(≤5.1)	Reference	Reference
Q1(5.1~6.0)	-0.025 (-0.105, 0.055)	0.498 (0.184, 1.343)
Q3(6.0~6.9)	-0.031 (-0.115, 0.053)	0.793 (0.365, 1.726)
Q4(>6.9)	0.070 (-0.002, 0.142)	1.777 (0.967, 3.267)
Females		
Q1(≤3.9)	Reference	Reference
Q2(3.9~4.7)	-0.028 (-0.110, 0.054)	2.271 (1.279, 4.032)
Q3(4.7~5.6)	0.045 (-0.030, 0.119)	3.197 (1.422, 7.189)
Q4(>5.6)	**0.124 (0.047, 0.201)**	**5.204 (2.148, 12.605)**

Data are expressed as odds ratio (95% Confidence interval [CI]).

Adjusted model including sex, age, race, smoking status, BMI, alcohol abuse, glycohemoglobin, systolic pressure, UACR, SCr, usage of diuretics and usage of pioglitazone. In the subgroup analyses of males and females, adjusted all confounders in the model except sex.

LSM, liver stiffness measurement; SCr, serum creatinine; UA, uric acid; UACR, albumin to creatinine ratio.

The bold indicates significance (P < 0.05).

## 4 Discussion

This study demonstrated that higher UA levels were independently associated with higher risk of liver steatosis and fibrosis in both males and females. In addition, the associations remained similar for females stratified by menopausal status. More importantly, to the best of our knowledge, this is the first study, performed in the United States nationally representative population, that employed VCTE to evaluate the association between UA with liver steatosis and fibrosis. Our current study illustrated that UA was positively associated with Ln CAP and Ln LSM, which suggested UA may be associated with the severity of liver steatosis and fibrosis.

In terms of the relationship between UA and liver steatosis, previous studies have showed different views. Lee K. has found that UA levels were independently associated with hepatic steatosis in a cross-sectional study of Korean adults (7). The research conducted by Zheng X et al. showed the positive associations between elevated UA levels and lean-NAFLD risk in Chinese adults (28). Another study showed that UA is a mediator of the association between obesity and incident NAFLD (29). Liver fatty disease was defined by abdominal ultrasonography in these studies and the results were consistent with ours. Compared with diagnosing liver steatosis with abdominal ultrasonography, VCTE was used in our study, which can quantify the degree of liver steatosis and liver stiff with accurate numbers. In this study, we found that UA was positively associated with Ln CAP, which suggested UA may be associated with the severity of liver steatosis. Conversely, in a recent study, carried out by Fan N, et al. and performed on 541 type 2 diabetic patients, showed that UA levels were not independently associated with the risk of NAFLD in female population (8). Another study showed that the presence of UA predicted the risk of NAFLD only in nonobese postmenopausal females, rather than in obese postmenopausal females (30). However, our study found the positive association of UA and liver steatosis held true for both premenopausal and postmenopausal females, which was consistent with an early study, showing that high levels of UA were associated with NAFLD both in premenopausal and postmenopausal females (31). The opposite results may result from that the study were focused on different patient populations.

Meanwhile, there were few large -sample size studies about the relationship between UA and liver fibrosis. One cross-sectional study including 1,365 NAFLD patients from Japan showed inverse correlation between UA levels and fibrosis stages (32). However, Afzali A et al. found that higher UA levels were associated with the increased hospitalization due to cirrhosis in a cohort study of USA participants (6). In a biopsy study of 166 NAFLD people, it was illustrated that high UA was directly related to NAFLD activity score (NAS) ≥ 5, which is an independent predictor of progression to advanced fibrosis (33, 34); it is reasonable to speculate that UA might participate in the progression of liver disease and the final cirrhosis development, by promoting and amplifying liver inflammation. Similarly, our current study with relatively large-sample size found that higher UA levels were associated with higher prevalence of liver fibrosis for both males and females in premenopausal or postmenopausal status, which means that timely reduction of uric acid levels may help prevent liver cirrhosis.

Validation in animals was performed by Xu CF et al. They conducted an experimental study to investigate the effect of hypouricemic therapy on the prevention of NAFLD in Mongolian gerbil; the degree of hepatic steatosis in the therapy group was significantly ameliorated (35). In addition, there were several studies focused on the related mechanisms. One study showed that Uric acid regulates hepatic steatosis and insulin resistance through the NLRP3 inflammasome (36). Another study showed that uric acid increases the hepatic stellate cells by activating inflammatory mediators including Toll-like receptor-4, Monocyte Chemoattractant Protein-1 and Cluster of Differentiation 68 mRNA expression in the liver, ultimately leading to liver fibrosis (37).

Besides, there are still some limitations of our study. First, due to the cross-sectional design, we cannot make a clear description of the cause-and-effect relationship. Further studies are required to reveal the causal relationship and possible mechanisms. Second, biopsy is the best way to confirm liver fatty degeneration and fibrosis, however it’s too hard to collect a large number of patients’ liver biopsies in an epidemiological study.

In conclusion, after adjusting for the potential confounders, higher UA levels were associated with higher prevalence of liver steatosis and fibrosis both in males and females. It implied that lowering UA levels might have a positive effect on the prevention of fatty liver disease and liver cirrhosis. However, more studies are still needed to confirm the causal relationship and the underlying mechanism.

## Data Availability Statement

The datasets presented in this study can be found in online repositories. The names of the repository/repositories and accession number(s) can be found below: https://www.cdc.gov/nchs/nhanes/index.htm.

## Ethics Statement

The studies involving human participants were reviewed and approved by National Center for Health Statistics Research Ethics Review Board. The patients/participants provided their written informed consent to participate in this study.

## Author Contributions

Study concept and design: HD and RZ; Acquisition of data: HD, RZ, XC, GY. Analysis and interpretation of data: HD, CS, YJ, and YH. Drafting of the manuscript: HD. critical revision of the manuscript for important intellectual content: JS and HW. Study supervision: JS and HW.

## Funding

This research is supported by the National Natural Science Foundation of China (82170800), Guangdong Basic and Applied Basic Research Foundation (2021A1515110682), Research initiation Project of Shunde Hospital of Southern Medical University (SRSP2021001).

## Conflict of Interest

The authors declare that the research was conducted in the absence of any commercial or financial relationships that could be construed as a potential conflict of interest.

## Publisher’s Note

All claims expressed in this article are solely those of the authors and do not necessarily represent those of their affiliated organizations, or those of the publisher, the editors and the reviewers. Any product that may be evaluated in this article, or claim that may be made by its manufacturer, is not guaranteed or endorsed by the publisher.

## Supplementary Material

Supplementary Table 1 Sensitivity analysis of association between CAP≥248dB/m, LSM≥7kPa, and UA
