# Supplementary material for: Associations of Uric Acid With Liver Steatosis and Fibrosis Applying Vibration Controlled Transient Elastography in the United States: A Nationwide Cross-Section Study
Source: Front Endocrinol (Lausanne). 2022 Jun 23;13:930224. doi: 10.3389/fendo.2022.930224 (PMC9259838; doi:10.3389/fendo.2022.930224)
Supplement: Supplementary Table 1 — Sensitivity analysis of association between CAP≥248dB/m, LSM≥7kPa, and UA [file DataSheet_1.docx]

Supplementary Material

# Supplementary Tables

**supplementary table1.** Sensitivity analyses of associations between CAP≥248dB/m, LSM≥7kPa, with UA

|  | CAP≥248dB/m | LSM≥7kPa |
| --- | --- | --- |
| SUA, mg/dL | OR (95%CI) | OR (95%CI) |
| model1 |  |  |
| total |  |  |
| Q1(≤4.4) | Reference | Reference |
| Q2(4.4~5.3) | 1.077 (0.681, 1.702) | **1.828 (1.083, 3.086)** |
| Q3(5.3~6.4) | **1.845 (1.039, 3.275)** | **1.698 (0.899, 3.207)** |
| Q4(>6.4) | **2.210 (1.351, 3.617)** | **2.174 (1.267, 3.731)** |
| males |  |  |
| Q1(≤5.1) | Reference | Reference |
| Q1(5.1~6.0) | **2.010 (1.142, 3.537)** | 0.864 (0.460, 1.620) |
| Q3(6.0~6.9) | 1.101 (0.567, 2.136) | 0.606 (0.324, 1.132) |
| Q4(>6.9) | **2.658 (1,348, 5.240)** | 1.420 (0.816, 2.469) |
| females |  |  |
| Q1(≤3.9) | Reference | Reference |
| Q2(3.9~4.7) | **1.719 (1.135, 2.604)** | 0.788 (0.353, 1.761) |
| Q3(4.7~5.6) | 1.584 (0.916, 2.738) | 1.800 (0.735, 4.409) |
| Q4(>5.6) | **3.095 (1.486, 6.445)** | 2.251 (0.941, 5.387) |

Data are expressed as odds ratio (95% Confidence interval [CI]).

Adjusted model included sex, age, race, smoking status, BMI, alcohol abuse, glycohemoglobin, systolic pressure, UACR, SCr, usage of diuretics and usage of pioglitazone. In the subgroup analyses of males and females, adjusted all confounders in the model except sex.

CAP, controlled attenuation parameter; LSM, liver stiffness measurement; SCr, serum creatinine; UA, uric acid; UACR, albumin to creatinine ratio.

The bold indicates significance (*P* < 0.05).

**supplementary table2.** The associations between UA with CAP in premenopausal and postmenopausal females

|  | Ln CAP | CAP≥274dB/m |
| --- | --- | --- |
| SUA, mg/dL | B (95%CI) | OR (95%CI) |
| Premenopausal females |  |  |
| Q1(≤3.7) | Reference | Reference |
| Q2(3.7~4.4) | 0.048 (-0.025, 0.121) | 1.956 (0.576, 6.640) |
| Q3(4.4~5.1) | 0.042 (-0.021, 0.105) | 2.206 (0.904, 5.382) |
| Q4(>5.1) | **0.128 (0.067, 0.188)** | **3.634 (1.613, 8.189)** |
| Postmenopausal females |  |  |
| Q1(≤4.2) | Reference | Reference |
| Q2(4.2~5.0) | **0.086 (0.017, 0.156)** | 1.813 (0.888, 3.705) |
| Q3(5.0~6.0) | **0.074 (0.002, 0.145)** | 2.354 (0.991, 5.589) |
| Q4(>6.0) | **0.091 (0.036, 0.146)** | 1.959 (0.841, 4.564) |

Data are expressed as odds ratio (95% Confidence interval [CI]).

Adjusted model included age, race, smoking status, BMI, alcohol abuse, glycohemoglobin, systolic pressure, UACR, SCr, usage of diuretics and usage of pioglitazone.

CAP, controlled attenuation parameter; SCr, serum creatinine; UA, uric acid; UACR, albumin to creatinine ratio.

The bold indicates significance (*P* < 0.05).

**supplementary table3.** The associations between UA with LSM in premenopausal and postmenopausal females

|  | Ln LSM | LSM≥8kPa |
| --- | --- | --- |
| SUA, mg/dL | B (95%CI) | OR (95%CI) |
| Premenopausal females |  |  |
| Q1(≤3.7) | Reference | Reference |
| Q2(3.7~4.4) | 0.014 (-0.069, 0.097) | 2.148 (0.397, 11.606) |
| Q3(4.4~5.1) | 0.048 (-0.031, 0.128) | 1.589 (0.293, 8.629) |
| Q4(>5.1) | **0.195 (0.108, 0.282)** | **6.670 (1.355, 32.836)** |
| Postmenopausal females |  |  |
| Q1(≤4.2) | Reference | Reference |
| Q2(4.2~5.0) | -0.020 (-0.127, 0.088) | **5.212 (1.858, 14.617)** |
| Q3(5.0~6.0) | 0.048 -0.053, 0.149) | 2.651 (0.996, 7.054) |
| Q4(>6.0) | 0.014 (-0.108, 0.136) | **4.613 (1.521, 13.986)** |

Data are expressed as odds ratio (95% Confidence interval [CI]).

Adjusted model included age, race, smoking status, BMI, alcohol abuse, glycohemoglobin, systolic pressure, UACR, SCr, usage of diuretics and usage of pioglitazone.

LSM, liver stiffness measurement; SCr, serum creatinine; UA, uric acid; UACR, albumin to creatinine ratio.

The bold indicates significance (*P* < 0.05).
